# Multimodal Neurorehabilitation Including Auditory EEG Neurofeedback: A Proof of Concept Tested After Resection of a Giant Olfactory Groove Meningioma

**DOI:** 10.3390/brainsci16101022

**Published:** 2026-09-25

**Authors:** Annamaria Romita, Rosaria Pia Spina, Nicolò Li Volsi, Claudia Cacciapaglia, Valerio Manippa, Maria Concetta Schiavariello, Leonardo Soleto, Pietro Fiore, Luca Speranzon, Stefania De Trane, Paolo Taurisano, Gianvito Lagravinese

**Affiliations:** 1 Laboratory of Neuropsychology, Istituti Clinici Scientifici Maugeri IRCCS, Bari Institute, 70124 Bari, Italy; 2 Department of Theoretical and Applied Sciences, eCampus University, 22060 Novedrate, Italy; valerio.manippa@uniecampus.it; 3 Neurorehabilitation Unit, Istituti Clinici Scientifici Maugeri IRCCS, Bari Institute, 70124 Bari, Italyleonardo.soleto@icsmaugeri.it (L.S.); pietro.fiore@icsmaugeri.it (P.F.); stefania.detrane@icsmaugeri.it (S.D.T.); 4 Department of Physical and Rehabilitation Medicine, University of Foggia, 71122 Foggia, Italy; 5 Division of Neurosurgery, Department of Translational Biomedicine and Neurosciences (DiBraiN), University “Aldo Moro” of Bari, 70124 Bari, Italy; luca.speranzon@policlinico.ba.it; 6 Department of Translational Biomedicine and Neuroscience, University of Bari Aldo Moro, 70124 Bari, Italy; paolo.taurisano@uniba.it

**Keywords:** olfactory groove meningioma, neurorehabilitation, EEG neurofeedback, cognitive rehabilitation, quantitative EEG, functional connectivity, executive dysfunction, case report

## Abstract

**Highlights:**

**What are the main findings?**

- A four-week multimodal rehabilitation program combining auditory EEG neurofeedback, individualized cognitive training, and physiotherapy was feasible in a patient with severe visual impairment after resection of a giant olfactory groove meningioma.
- Cognitive and functional improvement was accompanied by reduced theta activity, increased SMR and beta activity, an increased SMR/theta ratio, and frequency-specific changes in resting-state functional connectivity.

**What are the implications of the main findings?**

- Auditory neurofeedback and adapted cognitive training may be integrated into intensive postoperative neurorehabilitation when visual feedback is not clinically feasible.
- The findings are hypothesis-generating and support controlled studies designed to distinguish rehabilitation-related changes from surgery, spontaneous recovery, and other components of multimodal care.

**Abstract:**

**Background/Objectives:** Patients undergoing resection of large olfactory groove meningiomas may present with persistent cognitive, behavioral, motor, and functional impairments. Evidence regarding EEG neurofeedback as part of postoperative neurorehabilitation remains preliminary. This case report describes the feasibility and associated clinical and neurophysiological changes in a sensory-adapted multimodal rehabilitation program. **Methods:** A 53-year-old man underwent emergency resection of a giant olfactory groove meningioma and subsequently completed 20 inpatient rehabilitation sessions over four weeks. Each session included auditory SMR/theta EEG neurofeedback at Cz, individualized cognitive training, and physiotherapy. Neuropsychological, emotional, functional, and mobility outcomes were assessed before and after treatment. EEG outcomes included longitudinal single-channel recordings acquired during neurofeedback sessions and 19-channel resting-state recordings obtained under eyes-open and eyes-closed conditions before and after the program. Spectral power, spectral ratios, and weighted phase-lag index connectivity were analyzed descriptively. **Results:** Reliable or measurement-based improvements were observed in global cognition, executive functioning, immediate and delayed verbal recall, total and cognitive functional independence, and mobility. Across the 20 neurofeedback sessions, the conservative primary analysis retained 9936 of 10,261 epochs and showed reduced theta activity, increased SMR and beta activity, increased SMR/theta, and reduced theta/total-beta. Resting-state EEG showed lower relative theta power and higher SMR, beta-1, and beta-2 power under both recording conditions. Connectivity changes included reduced theta and posterior alpha coupling together with increased SMR connectivity across fronto-central and parieto-occipital networks. **Conclusions:** Sensory-adapted EEG neurofeedback was feasible within an intensive multimodal rehabilitation program and was accompanied by concurrent cognitive, functional, and neurophysiological changes. Because of the single-case, uncontrolled, multimodal design, the findings cannot establish treatment-specific efficacy and should be considered hypothesis-generating.

## 1. Introduction

Intracranial meningiomas are extra-axial tumors whose clinical impact may extend beyond focal neurological symptoms. When these lesions arise from the anterior skull base and reach considerable sizes, compression of the orbitofrontal and medial frontal regions, together with peritumoral edema and disruption of large-scale neural networks, may result in substantial cognitive, behavioral, and functional impairment. Patients with anterior skull base meningiomas may exhibit deficits in executive functioning, attention, processing speed, verbal fluency, working memory, and behavioral regulation, both before and after surgical treatment [1,2,3].

Although surgical resection may relieve mass effect and is frequently followed by improvements in neurological and cognitive functioning, postoperative recovery is heterogeneous. Prospective studies have shown that some patients continue to experience impairments in attention, cognitive flexibility, processing speed, and executive functioning even after apparently successful tumor removal [1,2,3]. This variability suggests that surgical and radiological outcomes alone may not fully capture recovery and highlights the importance of incorporating standardized neuropsychological and functional assessments into postoperative care.

Post-acute neurorehabilitation may therefore play an important role in promoting autonomy and addressing the combined motor, cognitive, and behavioral consequences of brain tumors and their treatment. Inpatient rehabilitation studies have documented meaningful functional gains following brain tumor surgery, while randomized controlled trials have shown that structured cognitive rehabilitation can improve domains such as attention, verbal memory, and everyday cognitive functioning [4,5,6]. Given the frequent coexistence of motor, sensory, and neuropsychological deficits, an individualized multimodal approach integrating physical therapy and cognitive training may be particularly appropriate. Nevertheless, detailed reports describing intensive integrated rehabilitation after resection of giant anterior skull base meningiomas remain limited.

Electroencephalography may complement clinical and neuropsychological measures by providing information on cortical oscillatory activity and functional network organization. Resting-state EEG characteristics have been associated with cognitive and language functioning in patients with gliomas and meningiomas, suggesting a potential role for EEG-derived measures in characterizing neurophysiological changes during recovery [7]. EEG neurofeedback extends this approach by providing real-time sensory information derived from the participant’s brain activity, thereby supporting the learned modulation of selected electrophysiological features. Although research regarding the use of neurofeedback for cognitive rehabilitation is still limited, the literature in this field is growing at a steady rate. For instance, in a randomized, double-blind, sham-controlled study involving patients with acquired frontal brain lesions, infra-low-frequency neurofeedback did not produce significantly greater cognitive improvement than sham training in the overall sample; however, exploratory analyses in patients with predominantly frontal lesions suggested possible improvements in frontal functioning and alertness [8]. Similarly, a prospective case series highlighting a combined neurofeedback and motor rehabilitation program on seven inpatients with post-brain tumor surgery cognitive deficits, reported normalization of pathologic delta/beta activity and cognitive improvements in all patients, especially regarding memory, executive functions and language [9]. These preliminary findings support the feasibility of neurofeedback in frontal neurorehabilitation while also emphasizing the need for cautious interpretation and further investigation.

Against this background, the present case report describes an exemplificative case of acquired brain injury secondary to a giant olfactory groove meningioma extending to the planum sphenoidale and chiasmatic region. The lesion was associated with severe bilateral visual field impairment related to compression of the optic pathways and with prominent behavioral and executive disturbances consistent with bilateral frontal-lobe compression. The patient underwent emergency surgical resection and subsequently participated in an intensive, 20-session inpatient neurorehabilitation program integrating auditory EEG neurofeedback, individually adapted cognitive training, and physical therapy. The intervention was adapted to the patient’s severe visual impairment and predominantly dysexecutive neuropsychological profile. The aim was to characterize the feasibility and tolerability of the integrated protocol and to describe the associated changes in neurological, functional, neuropsychological, and resting-state EEG outcomes. Given the single-case design and the simultaneous administration of multiple rehabilitation components, the study was not intended to isolate the specific contribution of neurofeedback or to establish a causal treatment effect, but rather to provide hypothesis-generating clinical and neurophysiological observations.

## 2. Materials and Methods

### 2.1. Case Presentation

On 25 May 2026, a 53-year-old right-handed man was admitted to the emergency department after being found unconscious at home with snoring respirations and unresponsive to verbal stimuli. The patient showed a medical history of keratoconus and an untreated depressive state over the two years following the death of his mother. He had completed 8 years of formal education and was regularly employed as a factory worker. At initial evaluation, he was assisted by a resuscitation specialist, scoring a 6/15 (E1, V1, M4) on the Glasgow Coma Scale (GCS), with absent pupillary response, medium mydriasis, and non-purposeful motor responses to painful stimuli. An arterial blood gas (ABG) test showed a balanced respiratory and metabolic profile.

An emergency contrast-enhanced whole-brain and spine computed tomography (CT) scan revealed a giant (65 mm × 65 mm × 50 mm, LL × AP × CC) isodense extra-axial olfactory groove mass with calcifications, extending to the planum sphenoidale and chiasmatic region, with marked bilateral frontal lobe compression and extensive peritumoral edema, as well as associated hyperostosis of the anterior skull base (Figure 1a,b).

Preoperative magnetic resonance imaging (MRI) was not available, as the patient was taken directly to the operating room for emergency life-saving surgery.

The lesion extended caudally to the suprasellar region, where it compressed the optic nerves and chiasm, consistent with the severe bilateral visual impairment observed clinically, and was associated with extensive bilateral fronto-parieto-temporal perilesional edema. Furthermore, it exerted marked mass effect on the rostrum and body of the corpus callosum, the frontal horns of the lateral ventricles, the third ventricle, the bilateral basal ganglia, the cerebral peduncles, and the midbrain, resulting in effacement of the basal cisterns and adjacent cortical sulci. The tumor also led to the posterior displacement of the anterior communicating and anterior cerebral arteries (ACoA-ACA complex) and was in close anatomical relation to the carotid siphons and cavernous sinuses. The left lateral ventricle displayed a reduction in volume, while the right lateral ventricle showed widening of the trigone and occipital horn.

Following neurological consultation, which attributed the comatose state to the mass lesion, the patient underwent an emergency bifrontal craniotomy for tumor resection, along with the cranialization of the frontal sinuses. An estimated extent of resection >98% was achieved; the procedure was classified as Simpson grade II. Histopathological examination confirmed a meningothelial meningioma, CNS WHO grade 1 according to the 2021 WHO classification. Immunohistochemical analysis showed positivity for epithelial membrane antigen (EMA) and progesterone receptor (PR), with negative CD34 staining. No mitotic activity was identified, and the Ki-67 proliferation index was approximately 2%.

On 26 May 2026 (postoperative day 1, POD 1), a non-contrast head CT scan showed expected postoperative changes, with evolution of the surgical findings and no evidence of active postoperative complications. An electroencephalogram (EEG) performed the same day showed generalized background slowing, characterized by theta-delta activity, slightly asymmetrical and with lower voltage in the left hemisphere, with no paroxysmal abnormalities indicative of status epilepticus.

On 27 May (POD 2), the unsedated patient kept his eyes closed and did not follow commands, but localized pain bilaterally and tended to flex his trunk.

On 31 May (POD 6), he was awake, cooperative, partially oriented, and able to execute simple commands with preserved motility in all four limbs.

On 3 June (POD 9), a follow-up EEG revealed alpha background activity that was occasionally poorly modulated but bilateral, synchronous, symmetrical, and partially reactive to eye opening.

On 10 June 2026, the patient was transferred to the Neuromotor Rehabilitation Unit 2 of the IRCCS Maugeri Institute for post-acute intensive neurorehabilitation.

Upon admission, neurological examination revealed generalized four-limb weakness (Medical Research Council [MRC] scale: 4/5), gait ataxia requiring moderate assistance with postural misalignment, and severe bilateral visual impairment.

On 16 June 2026, a postoperative head CT scan showed a reduction in bilateral frontal postsurgical edema compared with earlier postoperative imaging, together with evolution of the expected postoperative changes. The scan also showed a bifrontal extra-axial fluid collection (maximum thickness, 18 mm) containing fat tissue from the graft and small gas bubbles, and a 5-mm rightward midline shift. These radiological findings were observed in parallel with still partial but progressive clinical and neurological improvement (see Table 1 for Clinical Case Overview).

No additional neuroimaging was obtained after the June 16 CT during the subsequent four-week rehabilitation period, as the patient’s clinical condition was steadily improving and further CT imaging was not considered clinically necessary, thereby avoiding unnecessary exposure to ionizing radiation. Therefore, although a reduction in postoperative edema had been documented by June 16, its subsequent evolution during the rehabilitation intervention could not be quantified radiologically.

For clarity, the main postoperative milestones were as follows: emergency tumor resection on 25 May; persistence of severe neurological impairment on 27 May (POD 2), when the patient remained unable to follow commands; substantial early neurological improvement by May 31 (POD 6), when he was awake, cooperative, partially oriented, and able to execute simple commands; follow-up clinical EEG on 3 June (POD 9); transfer to the Neuromotor Rehabilitation Unit for post-acute intensive neurorehabilitation on 10 June; protocol-specific PRE resting-state EEG assessment on 16 June, immediately before initiation of the neurofeedback program; and completion of the 20-session rehabilitation program with POST resting-state EEG assessment on 17 July.

The patient’s primary caregiver was involved in the assessment process to provide collateral information regarding the patient’s cognitive, emotional, and behavioral functioning, as well as their level of independence in daily activities. The caregiver reported that, following the patient’s mother’s death several years earlier, the patient showed low mood and increased emotional blunting, although limited affective expression had always been present. Anxiety, mild disinhibition, irritability, emotional lability, and occasional verbal aggression—quickly subsiding when addressed—were also described. Despite poor organization, occasional need for prompting, and reduced self-care, the patient remained fully independent in daily activities. Socially, the patient had few friends but tended to initiate conversations indiscriminately, including with strangers in public settings. Taken together, these behavioral abnormalities were clinically consistent with dysfunction of frontal systems in the context of the extensive bilateral frontal-lobe involvement produced by the tumor and associated edema.

### 2.2. Neuromotor and Functional Assessment

At admission to the rehabilitation unit, the patient was awake, alert, conscious, and cooperative, although he was only partially oriented to space, time, and person. He presented ideomotor slowing, memory deficits, and a marked tendency toward perseveration. Comprehension was intact, and he was able to understand and follow simple commands; however, his spontaneous speech was distinctly tangential and repetitive.

Mobility was partially dependent at baseline: while the patient could perform changes in decubitus autonomously, he required moderate assistance for ambulation, exhibiting an ataxic gait with postural misalignment. Muscle tone was normal across all four limbs, yet bilateral quadriceps hypomyotrophy was evident. Upper limb palmar grips and lower limb strength against resistance were graded at 4/5 on the Medical Research Council (MRC) scale, consistent with generalized four-limb weakness in the context of prolonged postoperative immobilization and deconditioning.

Cranial nerve examination was generally unremarkable, with the notable exception of a severe bilateral visual impairment. Deep tendon reflexes were regular across all segments, superficial tactile sensation was preserved, and the plantar response was silent bilaterally. The surgical wounds were dry and in good condition, with sutures already removed, with no clinical signs of cerebrospinal fluid rhinorrhea. Systemically, the patient was afebrile, normotensive, and eupneic, although he experienced severe insomnia, reporting a complete absence of sleep during the initial days of hospitalization. Bladder and bowel continence were preserved. Functional performance was characterized by a high level of care complexity; the patient required 24-h nursing assistance and was admitted for intensive inpatient neurorehabilitation (Code 56) due to his unsuitability for outpatient management. During the rehabilitation period, levetiracetam was administered at 500 mg three times daily. Dexamethasone (Soldesam oral drops, 2 mg/mL [0.2%]) was progressively tapered, with 64 drops once daily administered for four days followed by 32 drops once daily for the subsequent four days. These medication changes were considered when interpreting the cognitive and electrophysiological PRE–POST findings.

### 2.3. Neuropsychological Assessment

Neuropsychological assessment was conducted at admission and repeated upon completion of the rehabilitation program to examine global cognitive functioning, attention and executive functions, verbal learning and memory, working memory, lexical access, visuospatial/constructional abilities, and emotional and behavioral functioning. Given the patient’s severe bilateral visual impairment, the assessment protocol preferentially relied on auditory and verbally mediated measures; visually demanding tasks were administered only when clinically feasible and were interpreted cautiously. Global cognitive functioning was assessed using the Mini-Mental State Examination (MMSE) [10]. Executive and attentional functioning was examined using the Frontal Assessment Battery (FAB) [11], Weigl’s Sorting Test, the Hayling Sentence Completion Test [12], and the Spatial Stroop Test. Verbal short-term and working memory were assessed using Digit Span Forward and Backward [13], whereas verbal learning and delayed recall were evaluated using the Rey Auditory Verbal Learning Test (RAVLT) [14]. Lexical retrieval was examined through phonemic and semantic verbal fluency tasks, while the Clock Drawing Test and Rey–Osterrieth Complex Figure were included among the visually mediated measures and administered when clinically feasible. Emotional and behavioral functioning was assessed using the Beck Depression Inventory (BDI), State-Trait Anxiety Inventory (STAI), Toronto Alexithymia Scale-20 (TAS-20) [15], and the Italian version of the Dimensional Apathy Scale (I-DAS) [16]. Where validated Italian normative corrections were available for the specific instrument and scoring system, raw scores were adjusted according to the demographic variables specified by the corresponding normative source and interpreted within that instrument’s normative framework. Corrected or equivalent scores were therefore used only when supported by the applicable standardization procedure rather than uniformly across all measures. The same assessment framework was used at the post-treatment evaluation to permit direct PRE–POST comparison. To minimize retest and practice effects, parallel or alternate forms were used at post-treatment whenever validated equivalent versions were available for the administered measure.

### 2.4. Clinical and Functional Outcome Measures

Clinical and functional status was assessed at admission to the rehabilitation program and reassessed upon program completion. Functional independence was evaluated using the Functional Independence Measure (FIM), considering the total score as well as the motor and cognitive subscores [17], and the Modified Barthel Index [18]. Functional mobility was assessed using the Timed Up and Go (TUG) test, with completion time expressed in seconds [19]. Pre–post changes in these measures were subsequently interpreted using the measurement-based criteria described in Section 2.7.1 [20,21,22]. The Trunk Control Test (TCT) was additionally recorded at admission to characterize baseline trunk control [23], whereas rehabilitation care complexity was characterized using the Italian Rehabilitation Complexity Scale–Extended (RCS-E) [24]. Because corresponding post-treatment values were not available for longitudinal comparison, these measures were used only for baseline clinical characterization and were not included in the pre–post change analyses.

### 2.5. Multimodal Rehabilitation Procedure

The patient underwent an intensive multimodal neurorehabilitation program consisting of 20 sessions delivered five days per week over four consecutive weeks. Each treatment day included 35 min of auditory EEG neurofeedback, 35 min of individualized cognitive training, and 35 min of physiotherapy, for a total planned duration of 105 min per session.

#### 2.5.1. Auditory EEG Neurofeedback

EEG neurofeedback was administered using a ProComp5 hardware system and BioGraph Infiniti software version 6.9.0.0 (Thought Technology Ltd., Montreal, QC, Canada), strictly adhering to standardized clinical biofeedback procedures. The active electrode was positioned at Cz, while reference and ground electrodes were placed on the patient’s ears, according to the International 10–20 System. Electrode impedances were maintained below 5 kΩ to guarantee high signal integrity. The protocol was specifically designed to promote cortical self-regulation by reinforcing the SMR frequency band (12–15 Hz) while concurrently inhibiting Theta (4–8 Hz) and EMG (52–58 Hz) activity. Each 35-min session utilized an auditory-based gamified interface—a “boat race” task—to foster patient engagement and provide immediate reinforcement for the targeted EEG dynamics. Due to the patient’s severe visual impairment, feedback was delivered primarily through this auditory modality. Reward and inhibit thresholds were manually adjusted in real time by the clinician throughout each session according to standard clinical neurofeedback procedures. The SMR reward threshold was calibrated to maintain an approximate reward success rate of 80%, whereas the theta and EMG inhibit thresholds were adjusted to maintain approximately 20% inhibit activation. As performance improved across sessions, thresholds were progressively recalibrated to preserve this 80/20 reinforcement contingency and maintain an appropriate level of task difficulty while minimizing ceiling effects. Because these success percentages were intentionally constrained by adaptive clinician-controlled thresholding, they were not treated as independent indices of neurofeedback learning. Instead, training-related electrophysiological modulation was examined descriptively through the offline longitudinal analysis of the recorded EEG signal across all 20 sessions. The patient’s adherence and potential adverse effects were monitored throughout the protocol.

#### 2.5.2. Cognitive Training

Integrated cognitive training was delivered using the Khymeia system (version 5.2.32.16055), focusing on short-term and working memory, verbal learning, delayed recall, sustained and selective attention, processing speed, response inhibition, and strategic problem solving. Task difficulty was progressively adjusted according to the patient’s accuracy and response time, with advancement to the next level contingent upon a predefined success criterion in the previous session. The training program comprised several core modules, beginning with the “Safe” (Cassaforte) module, which was utilized to challenge short-term and working memory capacity. In this task, the patient was required to memorize and subsequently recall sequences of increasing length, performing both forward and backward order retrieval; by requiring the patient to hold and manipulate numerical or symbolic sequences, this module stressed the phonological loop and the central executive’s ability to maintain and reorganize information in real-time, thereby fostering higher-order executive control [25].

Mnemonic traces were further stimulated through the “Word Memorization” module, which was employed to facilitate the encoding, consolidation, and retrieval of information. To circumvent the patient’s visual impairments, this exercise was adapted to an auditory-only modality where the clinician read aloud a target list of words for the patient to memorize, followed by a subsequent list containing target words mixed with distractors. This task was designed to progressively increase in linguistic complexity, incorporating phonologically or semantically similar words to force the patient to engage active rehearsal strategies to bridge the gap between initial perception and delayed recall. Furthermore, visual memory training modules were implemented to enhance visuospatial retention and recall, utilizing high-contrast, color-coded geometric symbols to accommodate sensory constraints. The task difficulty was progressively scaled through an increasing number of card pairs, encouraging the patient to shift from random selection to the utilization of logical, structured elimination and spatial mapping strategies. A specific narrative comprehension module was also implemented, requiring the patient to listen to and retain narrative passages of increasing length and complexity. This training was structured to systematically increase text length and question complexity, with higher levels incorporating inquiries regarding fine details of the narrative. To assess consolidation mechanisms, a delayed recall task was administered after a 10-min interval, requiring the patient to maintain and retrieve verbal information over time.

Finally, attentional control and processing speed were refined through “Attentional Matrices,” which compelled the patient to filter out distractors and maintain focus on specific high-contrast, color-coded geometric symbols, and through dedicated “Reaction Time” tasks designed to optimize cortical processing speed and response inhibition by requiring the patient to suppress impulsive motor actions in favor of accurate, time-sensitive execution [26].

#### 2.5.3. Physiotherapy

The rehabilitation program was supported by daily conventional physiotherapy aimed at maximizing functional autonomy. Motor interventions focused on clinical stabilization, core and lower limb muscle strengthening, postural control, and gait management. The physiotherapy sessions included specific training drills to address static and dynamic balance, as well as the management of ataxia. Exercises were systematically adapted to accommodate the patient’s sensory constraints, utilizing specific verbal cues and tactile feedback to facilitate movement execution and ensure safety during postural transitions and walking. The intensity and complexity of the motor tasks were titrated based on the patient’s physical fatigue and clinical progression throughout the duration of the program.

### 2.6. EEG Acquisition and Data Processing

EEG data were obtained through two complementary acquisition streams. First, multichannel resting-state EEG was recorded before and after completion of the rehabilitation program under eyes-open and eyes-closed conditions to characterize changes in spectral activity and sensor-level functional connectivity. Second, continuous single-channel EEG was recorded from Cz during each of the 20 neurofeedback sessions to examine longitudinal within-treatment spectral trajectories. All offline analyses were performed in MATLAB R2025b (MathWorks, Natick, MA, USA) using EEGLAB version 2026.0.0 [27].

#### 2.6.1. Resting-State EEG Acquisition and Processing

Protocol-specified resting-state EEG assessments were conducted at two time points: on 16 June 2026, immediately before initiation of the 20-session neurofeedback program (PRE), and on 17 July 2026, following completion of the final neurofeedback session (POST). These recordings were acquired specifically for quantitative PRE–POST electrophysiological analyses and were not separately interpreted as routine clinical EEG examinations. EEG was recorded using a clinical 19-channel Mitsar/WinEEG system at a sampling rate of 250 Hz. Electrodes were positioned according to the international 10–20 system at Fp1, Fp2, F7, F3, Fz, F4, F8, T7, C3, Cz, C4, T8, P7, P3, Pz, P4, P8, O1, and O2. At both assessment time points, recordings were obtained under eyes-open and eyes-closed resting-state conditions. During each condition, the patient was instructed to remain awake, relaxed, and as still as possible, without performing an active cognitive task. The four recordings were exported in European Data Format and processed using a fully scripted and standardized pipeline. The original legacy labels T3, T4, T5, and T6 were renamed T7, T8, P7, and P8, respectively, and standard 10–5 template coordinates distributed with EEGLAB were assigned to the electrodes. To obtain an identical nominal recording length across conditions, the interval from 10 to 310 s was selected from each recording, yielding a common 300-s analysis segment before artifact removal. Signals were band-pass filtered between 1 and 80 Hz using a zero-phase finite impulse response filter, followed by a 48–52 Hz band-stop filter. Persistent channel noise was evaluated using the channel root mean square amplitude, robust standard deviation, ratio of 30–80 Hz to 1–30 Hz power, mean absolute correlation with the remaining montage, and percentage of near-zero first temporal differences. Robust z scores were calculated across channels using the median and median absolute deviation. A channel was marked for removal when at least one of the following criteria was met: (i) a robust z score greater than 5 for root mean square amplitude together with a mean absolute interchannel correlation below 0.30; (ii) a robust z score greater than 4 for the high-frequency power ratio together with a mean correlation below 0.40; (iii) a robust z score greater than 3 for the high-frequency power ratio together with a mean correlation below 0.33; (iv) a robust standard deviation below 10% of the montage median; or (v) near-zero first differences in more than 1% of samples. A maximum of three channels could be removed from an individual recording. Gross transient artifacts were screened using consecutive non-overlapping 2-s intervals. Broadband power between 1 and 45 Hz was calculated for every channel and interval, and the logarithm of the median broadband power across channels was standardized using a robust z score. Intervals with a robust z score greater than 3.5 were removed before independent component analysis. The remaining channels were re-referenced to their common average and decomposed using extended Infomax independent component analysis [28]. Principal component reduction was used to match the decomposition dimensionality to the theoretical data rank following average referencing. Independent components were classified using ICLabel [29]. Components were automatically removed when the estimated probability of an ocular source was at least 0.60 or the probability of a muscular source was at least 0.75. Previously removed channels were subsequently reconstructed using spherical interpolation, and the complete 19-channel dataset was re-referenced to the common average. For quantitative PRE–POST comparisons, interpolated electrodes were excluded to prevent reconstructed signals from contributing to scalar spectral estimates or functional-connectivity measures. Accordingly, eyes-open quantitative analyses were performed on 17 matched non-interpolated channels after exclusion of Fp2 and P7, whereas eyes-closed analyses were performed on 18 channels after exclusion of P7. All 19 channels, including interpolated electrodes, were retained exclusively for continuous scalp-topography visualization. Power spectral density was estimated separately for each channel using Welch’s method [30]. The continuous cleaned signal was linearly detrended and divided into 2-s Hann-windowed segments with 50% overlap. A 1024-point fast Fourier transform was used, corresponding to a frequency resolution of approximately 0.244 Hz. Absolute spectral power was obtained by numerical integration of the power spectral density within the following non-overlapping frequency ranges: delta, 1–4 Hz; theta, 4–7.5 Hz; alpha, 8–12 Hz; sensorimotor rhythm (SMR), 12–15 Hz; beta-1/low-beta, 15–20 Hz; beta-2/high-beta, 20–29 Hz; and low-gamma, 29–45 Hz. Frequency intervals were implemented as lower-bound inclusive and upper-bound exclusive, except for low-gamma, which included 45 Hz. The 7.5–8 Hz interval was retained in the total-power denominator but was not assigned to a specific band. Total power was calculated between 1 and 45 Hz, and relative band power was expressed as the proportion of total 1–45 Hz power accounted for by each frequency band. Two spectral ratios were additionally calculated: theta/total-beta, defined as theta power divided by the sum of beta-1 and beta-2 power, and SMR/theta. For regional analyses, electrodes were grouped as follows: frontal, Fp1, Fp2, F7, F3, Fz, F4, and F8; central, C3, Cz, and C4; temporal, T7, T8, P7, and P8; parietal, P3, Pz, and P4; and occipital, O1 and O2. Regional spectral values were calculated by averaging the available non-interpolated electrodes within each region. Sensor-level functional connectivity was estimated using the weighted phase-lag index (wPLI), which emphasizes consistently non-zero phase-lagged interactions and reduces sensitivity to instantaneous coupling associated with common sources and volume conduction [31]. For every electrode pair and frequency band, the imaginary component of the cross-spectrum was obtained across Welch windows and frequency bins. Band-level wPLI was calculated as the absolute mean of the imaginary cross-spectrum divided by the mean of its absolute value. Connectivity results were summarized separately for within-region and between-region electrode pairs. Reported connectivity analyses included theta, alpha, SMR, beta-1, beta-2, and low-gamma; delta-band connectivity was not interpreted. Any electrode pair containing an interpolated channel was excluded. A complementary sensitivity analysis was conducted using consecutive non-overlapping 2-s epochs. Epochs with a robust z score greater than 3.5 for the logarithm of median 1–45 Hz broadband power were excluded, and band-power and spectral-ratio outcomes were summarized using the median across the remaining epochs. This analysis was used to determine whether the direction and magnitude of the principal PRE–POST findings were robust to an alternative epoch-based summarization procedure.

#### 2.6.2. Single-Channel EEG Acquisition and Offline Spectral Analysis During Neurofeedback

Continuous single-channel EEG data were recorded throughout each of the 20 neurofeedback sessions using the ProComp5 system and BioGraph Infiniti software version 6.9.0.0 (Thought Technology Ltd., Montreal, QC, Canada). The Cz signal described above was sampled at 256 Hz and subsequently exported from BioGraph Infiniti as semicolon-delimited text files for offline analysis. Signal processing was performed in MATLAB R2025b (MathWorks, Natick, MA, USA) using EEGLAB version 2026.0.0. Given the single-channel recording configuration, procedures requiring multichannel spatial information, including independent component analysis, average rereferencing, bad-channel interpolation, and spatial artifact correction, were not applicable. Artifact management was therefore performed at the epoch level. Each continuous recording was band-pass filtered between 1 and 48 Hz using a zero-phase finite impulse response filter. To limit edge effects, the first and last 5 s of each filtered recording were discarded. The remaining signal was divided into consecutive, non-overlapping 4-s epochs. For each epoch, peak-to-peak amplitude, maximum absolute amplitude, standard deviation, root mean square amplitude, and root mean square of the first temporal derivative were calculated. Robust z scores were obtained using the median and median absolute deviation across epochs within each session.

In the conservative primary analysis, an epoch was excluded when at least one of the following criteria was met: peak-to-peak amplitude greater than 100 µV, maximum absolute amplitude greater than 75 µV, standard deviation lower than 0.25 µV, or an absolute robust z score greater than 3.5 for peak-to-peak amplitude, root mean square amplitude, or first-derivative root mean square. Rejected epochs were omitted from subsequent spectral analyses without interpolation or temporal concatenation. To evaluate the robustness of the findings to the artifact-rejection thresholds, the complete analysis was repeated using a secondary, less restrictive procedure. In this analysis, epochs were excluded when peak-to-peak amplitude exceeded 150 µV, maximum absolute amplitude exceeded 100 µV, standard deviation was lower than 0.25 µV, or the absolute robust z score exceeded 5 for peak-to-peak amplitude, root mean square amplitude, or first-derivative root mean square. All remaining preprocessing and spectral-estimation parameters were unchanged. Power spectral density was estimated separately for each retained epoch using Welch’s method, with a 512-sample Hann window, 256-sample overlap, and a 1024-point fast Fourier transform, corresponding to a frequency resolution of 0.25 Hz. Absolute band power was calculated by numerical integration of the power spectral density within the following predefined frequency ranges: delta, 1–4 Hz; theta, 4–7.5 Hz; alpha, 8–12 Hz; sensorimotor rhythm (SMR), 12–15 Hz; beta-1/low-beta, 15–20 Hz; beta-2/high-beta, 20–29 Hz; and low-gamma, 29–45 Hz. Total spectral power was calculated between 1 and 45 Hz. The 7.5–8 Hz interval was retained in the total-power denominator but was not assigned to any predefined frequency band. Relative band power was expressed as the percentage contribution of each frequency band to total 1–45 Hz power. Three spectral ratios were additionally calculated for each epoch: SMR/theta; theta/total-beta, defined as theta power divided by the sum of beta-1 and beta-2 power; and SMR/beta-2. For each session, absolute power, relative power, and spectral-ratio values were summarized using the median across all retained epochs.

### 2.7. Statistical Analyses

Clinical, neuropsychological, functional, and longitudinal neurofeedback-session EEG outcomes were analyzed at the individual-patient level using descriptive and measurement-based change indices.

#### 2.7.1. Clinical and Neuropsychological Change

Pre–post changes were evaluated at the individual-patient level using formal change indices only when psychometric parameters were available for the same instrument and a sufficiently comparable scoring system. For the Frontal Assessment Battery (FAB), the reliable change index (RCI) was calculated according to Jacobson and Truax [32] as the pre–post difference divided by the standard error of the difference. The latter was derived from the normative standard deviation (SD = 1.8) and test–retest reliability coefficient (r = 0.85) reported in the Italian validation by Appollonio et al. [11]; absolute RCI values of 1.96 or greater were considered statistically reliable. For the immediate and delayed recall scores of the parallel 15-word lists, change was quantified using the ZD method described by Carlesimo et al. [14]. Values outside the published tolerance interval of ±1.65 were considered reliable. The primary calculation followed the A-to-B order documented in the data file, and a sensitivity analysis examined every possible ordered pair of the three parallel forms.

Measurement-based clinical significance was examined separately from statistical reliability. The MMSE change was compared with the published 95% minimal detectable change (MDC95) of 2.84 points [33]. Functional outcomes were interpreted using the published minimal clinically important differences (MCIDs) of 22, 17, and 3 points for the total, motor, and cognitive FIM scores, respectively [20]. For the Modified Barthel Index, the individual change threshold was calculated as 19% of the mean of the pre- and post-treatment scores, in accordance with the percentage-based MDC recommended in the presence of heteroscedastic measurement error [21]. For the Timed Up and Go test, the original 95% smallest real difference limits were applied, with a post-minus-pre change below −3.75 s indicating reliable improvement [22].

Because the MMSE, FIM, Modified Barthel Index, and Timed Up and Go benchmarks were derived from community-dwelling or stroke samples rather than patients with brain tumors, they were treated as indirect supportive criteria. For the remaining measures, RCI, MCID, or MDC values were not calculated when the exact version, scoring system, or directly applicable test–retest parameters could not be verified; these outcomes were therefore reported descriptively. TAS-20 category transitions were interpreted using the established Italian classification [15], whereas I-DAS subscores were described separately because opposing domain trajectories can be obscured by the total score [16]. No adjustment for multiple comparisons was applied. Given the single-case, multimodal design, all analyses were descriptive and hypothesis-generating and were not used to attribute change specifically to neurofeedback.

#### 2.7.2. Resting-State EEG Analysis

Resting-state EEG outcomes were examined descriptively at the individual-patient level. For each eyes-open and eyes-closed condition, whole-scalp and regional PRE–POST changes were calculated for absolute band power, relative band power, theta/total-beta, SMR/theta, and regional wPLI. Percentage change was calculated as [(POST − PRE)/PRE] × 100.

Because proportional changes in wPLI may become unstable when baseline connectivity approaches zero, connectivity findings were primarily reported as absolute POST-minus-PRE differences.

No inferential hypothesis testing was performed because all observations originated from a single patient and individual channels, frequency bands, and regional connections could not be considered statistically independent observations. Sensitivity-analysis agreement was evaluated using the proportion of regional outcomes showing the same direction of change as the primary analysis and the descriptive correlation between the corresponding percentage-change estimates. These coefficients were treated exclusively as indices of concordance and were not subjected to significance testing.

Low-gamma power and connectivity were treated as exploratory outcomes because high-frequency scalp EEG may retain residual sensitivity to cranial and facial muscle activity even after channel rejection and independent-component correction.

#### 2.7.3. Longitudinal Analysis of Neurofeedback-Session EEG

The longitudinal EEG analysis was descriptive and exploratory. The session-level median values obtained from epochs retained by the conservative primary artifact-rejection procedure were treated as the units of analysis, thereby assigning equal weight to each neurofeedback session irrespective of differences in recording duration. 

To facilitate visualization of changes across the four-week intervention, the 20 sessions were grouped into four consecutive five-session blocks: sessions 1–5, 6–10, 11–15, and 16–20. For each block, relative band-power and spectral-ratio outcomes were summarized as the mean and standard deviation of the five-corresponding session-level medians.

The principal longitudinal outcomes were relative delta, theta, alpha, SMR, beta-1, beta-2, and low-gamma power, together with the SMR/theta, theta/total-beta, and SMR/beta-2 ratios. No inferential hypothesis testing was performed because all observations originated from a single participant.

A secondary sensitivity analysis repeated the complete pipeline using the less restrictive artifact-rejection thresholds described above. This analysis was used exclusively to determine whether the direction and overall shape of the longitudinal trajectories depended on the conservative primary rejection criteria.

## 3. Results

### 3.1. Clinical, Neuropsychological, and Functional Outcomes

Following the four-week multimodal program, gains were observed across global cognition, executive functioning, verbal learning and memory, and several additional neuropsychological measures (Table 2). The MMSE increased from 23 to 29 (+6), exceeding the published MDC95 of 2.84 points. The FAB increased from 5 to 17 (+12), yielding an RCI of 12.17 and therefore exceeding the conventional threshold for statistically reliable improvement. RAVLT/RWL immediate recall increased from 26 to 41 (ZD = 3.27), and delayed recall increased from 4 to 8 (ZD = 2.98); both values exceeded the ±1.65 tolerance limits. Sensitivity analyses confirmed that both improvements remained reliable under every possible ordered pairing of the three parallel forms. Favorable raw score changes were also observed for the Clock Drawing Test, immediate and delayed prose recall, Digit Span Forward and Backward, Weigl’s Sorting Test, phonemic and semantic fluency, and Spatial Stroop completion time and errors. These changes were interpreted descriptively because directly applicable longitudinal psychometric parameters could not be verified for the specific versions and scoring systems used. Marked descriptive improvement was also observed on the Hayling Sentence Completion Test. Section A completion time decreased from 106.53 to 40.35 s, whereas Section B completion time decreased from 214.35 to 134.23 s. In parallel, Section B total errors decreased from 10/15 to 2/15 and correct responses increased from 5/15 to 13/15. According to the normative interpretation of the Italian version, performance shifted from markedly impaired at baseline to within the normative range at post-treatment assessment, indicating substantial improvement in processing speed and verbal response inhibition [12].

Functional independence and mobility also improved (Table 3). The total FIM score increased from 80 to 110 (+30), exceeding the 22-point MCID, and the cognitive FIM score increased from 15 to 30 (+15), exceeding the 3-point MCID. The motor FIM score increased from 65 to 80 (+15), but did not reach the published 17-point MCID. The Modified Barthel Index increased from 66 to 72 (+6); however, this change was smaller than the participant-specific MDC threshold of 13.11 points and therefore did not exceed measurement error. TUG performance improved from 18.35 to 9.11 s, corresponding to a reduction of 9.24 s. This change exceeded the original 95% smallest real difference limit for improvement (−3.75 s), indicating reliable improvement in functional mobility. Because these benchmarks were derived from stroke cohorts, they were interpreted as indirect supportive criteria in the present neuro-oncological case.

Emotional and behavioral outcomes showed mixed descriptive trajectories (Table 4). The BDI decreased from 9 to 0, and the TAS-20 decreased from 66 to 39, shifting from the alexithymic to the non-alexithymic range. STAI-Y1 and STAI-Y2 increased slightly from 36 to 40 and from 38 to 40, respectively. The I-DAS total score increased from 26 to 32; the executive-apathy subscore decreased from 14 to 3, whereas the emotional and initiation subscores increased from 9 to 13 and from 3 to 16, respectively. These changes were not classified as reliable or clinically important because no directly applicable change thresholds were identified. Given the markedly divergent subscale trajectories, the increase in the I-DAS total score was not interpreted as evidence of a uniform worsening of apathy.

### 3.2. Longitudinal EEG Dynamics During Neurofeedback Sessions

Across the 20 neurofeedback sessions, 10,261 non-overlapping 4-s epochs were available for analysis. Application of the conservative primary artifact-rejection criteria resulted in the exclusion of 325 epochs (3.17%), leaving 9936 epochs for spectral analysis. All sessions retained a substantial amount of usable EEG data and were therefore included in the longitudinal analysis.

Descriptive analysis across consecutive five-session blocks revealed a progressive redistribution of relative spectral power (Figure 2A). Between the first and final five-session blocks, relative theta power decreased by 35.3%, whereas relative SMR power increased by 10.1%. Relative beta-2 power increased by 50.1%, and relative low-gamma power increased by 34.5%. Relative delta power also showed an overall reduction across the intervention, while relative alpha power remained comparatively stable. Relative beta-1 power showed an overall increase.

Spectral-ratio trajectories were consistent with a shift away from slower-frequency predominance (Figure 2B). Between the first and final blocks, the SMR/theta ratio increased by 63.4%, whereas the theta/total-beta ratio decreased by 54.0%. The SMR/beta-2 ratio also decreased across blocks, reflecting the larger proportional increase in beta-2 activity relative to SMR.

High-frequency findings, particularly those involving low-gamma activity, were regarded as exploratory because residual myogenic contributions cannot be completely excluded from single-channel scalp EEG.

The secondary, less restrictive analysis excluded 111 of the 10,261 available epochs (1.08%), leaving 10,150 epochs for spectral analysis. The resulting trajectories were closely comparable with those obtained using the conservative primary procedure and showed the same overall pattern of reduced theta activity, increased SMR and beta activity, increased SMR/theta, and reduced theta/total-beta. This concordance indicated that the principal descriptive findings were not dependent on the conservative artifact-rejection thresholds.

### 3.3. Cognitive Training Outcomes

The analysis of daily performance data extracted from the Khymeia computerized system across the 20 rehabilitation sessions showed progressive improvement across the trained tasks. To facilitate interpretation of temporal trends, the sessions were analyzed both continuously and by grouping them into four consecutive 5-day blocks. Within-training performance gains were particularly evident in the verbal span tasks, in which progressively higher levels were reached across the four-week period.

Specifically, adapting to the patient’s severe visual impairment, the span tasks were administered verbally: the clinician read aloud numerical sequences that the patient was required to repeat orally, in either forward or backward order. Over the course of the 20 days, performance showed a gradual improvement as the difficulty level systematically increased. The Forward Span expanded from an initial baseline of 2 items on Day 1 to a stable 7 items by the final sessions, while the Backward Span improved from 2 to 5–6 items, demonstrating progressive improvement on the trained verbal span tasks (Figure 3b).

Performance also improved across the trained Attentional Matrices and Reaction Time tasks. Execution times for the Attentional Matrices dropped markedly, decreasing from over 5 min during the initial phase to just 0.52 min on Day 20 (Figure 3c). Performance on Reaction Time tasks exhibited a similar progressive reduction in execution time, dropping from an initial average of approximately 3 min to roughly 1.3 min by the end of the training (Figure 3c). Crucially, as the clinician continuously escalated the difficulty level for the Reaction Time module from level 1 to level 12 (Figure 3d), the patient successfully maintained a near-perfect accuracy score, consistently ranging between 96 and 100 throughout the entire protocol (see Figure 3a).

Progressive within-training performance changes were also observed in the verbal and visual memory modules, with the patient advancing to higher difficulty levels while maintaining relatively high accuracy. The patient successfully accommodated the progressive escalation in task difficulty, advancing from level 1 to level 8 in Word Memorization and reaching level 11 in Visual Memory (Figure 3d). Long-Term Memory (MLT) tasks similarly saw a progression up to level 4 (Figure 3d). Despite the higher cognitive loads imposed by these advanced levels, accuracy scores for word memorization and long-term memory remained highly stable, primarily ranging between 75 and 100 (Figure 3a). Overall, these trajectories indicate learning and improved performance on the trained tasks. Because the same task families were practiced repeatedly across sessions, these within-training gains should not be interpreted independently as evidence of generalized cognitive recovery or transfer to untrained cognitive functions. Broader cognitive change was assessed separately through the pre–post neuropsychological evaluation described in Section 3.1.

### 3.4. Resting-State qEEG Outcomes

All four resting-state recordings successfully completed the standardized preprocessing procedure. The initial analysis interval comprised 300 s per recording. In the eyes-open PRE recording, one 2-s interval showing excessive broadband power was removed, leaving 298 s of data; the remaining recordings retained the complete nominal 300-s interval. P7 was identified as persistently noisy in all four recordings, while Fp2 was additionally removed from the eyes-open PRE recording. These channels were spherically interpolated after independent-component correction.

No independent component met the predefined removal thresholds in the eyes-open PRE recording. In the eyes-open POST recording, four components were removed, comprising two ocular and two muscular components. One ocular component was removed from the eyes-closed PRE recording, and one muscular component was removed from the eyes-closed POST recording. All final datasets contained 19 channels, showed no non-finite values, and demonstrated numerically appropriate common-average referencing. Quantitative spectral and connectivity summaries were subsequently calculated using the matched non-interpolated channel sets described above.

#### 3.4.1. Spectral Power and Ratios

Resting-state EEG showed a marked PRE–POST redistribution from slower toward sensorimotor and faster-frequency activity under both recording conditions (Table 5 and Figure 4).

During eyes-open recording, absolute delta, theta, and alpha power decreased by 34.6%, 47.1%, and 36.8%, respectively. In contrast, absolute SMR increased by 14.9%, beta-1 by 25.0%, beta-2 by 73.2%, and low-gamma by 74.4%. Relative-power findings showed the same general pattern. Relative theta power decreased from 19.79% to 12.85% of total 1–45 Hz power, corresponding to a 35.1% reduction, while relative alpha decreased from 21.89% to 17.36% and relative delta from 23.90% to 19.52%. Relative SMR increased from 6.26% to 9.46%, beta-1 from 8.79% to 13.93%, beta-2 from 5.64% to 11.80%, and low-gamma from 4.09% to 8.72%. These changes were also reflected in the composite spectral ratios. The eyes-open theta/total-beta ratio decreased from 1.715 to 0.536, corresponding to a 68.7% reduction, whereas the SMR/theta ratio increased from 0.333 to 0.799, corresponding to a 140.2% increase. The eyes-closed recording showed an even larger redistribution. Absolute delta power decreased by 30.6%, theta by 59.2%, and alpha by 14.3%. Absolute SMR increased by 105.5%, beta-1 by 128.2%, beta-2 by 253.7%, and low-gamma by 332.5%. Relative theta power decreased from 22.85% to 9.32%, while relative delta decreased from 23.15% to 16.93% and relative alpha from 23.83% to 19.21%. Relative SMR increased from 5.00% to 9.78%, beta-1 from 6.76% to 13.93%, beta-2 from 4.56% to 13.26%, and low-gamma from 3.52% to 11.52%. The eyes-closed theta/total-beta ratio decreased from 2.507 to 0.380, corresponding to an 84.9% reduction. Conversely, the SMR/theta ratio increased from 0.225 to 1.144, representing a 407.5% increase. Topographically, the reduction in theta activity was widespread but was particularly evident across posterior and temporal regions. Increases in SMR were most apparent over frontal, central, and parietal areas, whereas beta-2 and low-gamma increases showed a broader central, parietal, and occipital distribution. The proportional low-gamma increases were large but were interpreted as exploratory because residual myogenic contributions cannot be completely excluded at scalp level.

#### 3.4.2. Sensor-Level Functional Connectivity

Regional wPLI analyses revealed frequency- and condition-specific PRE–POST changes rather than a uniform global increase or decrease in connectivity (Table 6 and Figure 5).

During eyes-open recording, mean within-temporal theta connectivity decreased from 0.267 to 0.063 (ΔwPLI = −0.204), while temporal–parietal theta connectivity decreased from 0.171 to 0.048 (ΔwPLI = −0.123). In contrast, SMR connectivity increased between parietal and occipital regions from 0.068 to 0.216 (ΔwPLI = +0.147) and between frontal and occipital regions from 0.050 to 0.158 (ΔwPLI = +0.108). Low-gamma connectivity increased within temporal regions from 0.035 to 0.189 (ΔwPLI = +0.154), within central regions from 0.074 to 0.203 (ΔwPLI = +0.129), and between central and temporal regions from 0.039 to 0.148 (ΔwPLI = +0.109).

During eyes-closed recording, alpha connectivity decreased substantially between the two occipital electrodes, from 0.283 to 0.005 (ΔwPLI = −0.278), as well as within parietal regions, from 0.240 to 0.074 (ΔwPLI = −0.166), and within central regions, from 0.220 to 0.075 (ΔwPLI = −0.144). Theta connectivity decreased within parietal regions from 0.208 to 0.043 (ΔwPLI = −0.164), between temporal and parietal regions from 0.195 to 0.049 (ΔwPLI = −0.146), and within temporal regions from 0.214 to 0.089 (ΔwPLI = −0.126). Conversely, eyes-closed SMR connectivity increased between parietal and occipital regions from 0.079 to 0.281 (ΔwPLI = +0.202), between frontal and occipital regions from 0.043 to 0.220 (ΔwPLI = +0.177), between central and parietal regions from 0.102 to 0.215 (ΔwPLI = +0.114), and between frontal and central regions from 0.067 to 0.165 (ΔwPLI = +0.098). Overall, the connectivity findings were characterized by reduced theta and posterior alpha phase coupling together with increased SMR coupling across fronto-central and parieto-occipital networks. High-frequency connectivity changes were more prominent during eyes-open recording and were considered exploratory.

#### 3.4.3. Sensitivity Analysis

The epoch-based sensitivity analysis provided strong descriptive support for the principal spectral findings. Of the 160 regional band-power and spectral-ratio comparisons, 154 showed the same direction of change in the primary and sensitivity analyses, corresponding to 96.3% directional agreement. Percentage-change estimates showed a high descriptive concordance between the two approaches, with a Pearson correlation of r = 0.91 and a Spearman correlation of ρ = 0.97. These coefficients were not subjected to inferential testing because the regional and frequency-specific observations were not statistically independent. The few discordant findings were confined to six regional metrics and did not alter the overall pattern of theta reduction, increased SMR and beta activity, reduced theta/total-beta, and increased SMR/theta.

## 4. Discussion

This case report describes the clinical, cognitive, functional, and neurophysiological evolution of a patient who underwent emergency resection of a giant olfactory groove meningioma and subsequently completed a four-week multimodal neurorehabilitation program combining auditory EEG neurofeedback, individualized cognitive training, and physiotherapy (Table 2, Table 3, Table 4, Table 5 and Table 6; Figure 2, Figure 3, Figure 4 and Figure 5). The protocol was completed despite severe bilateral visual impairment and an initially prominent dysexecutive syndrome, supporting the feasibility of an intensive rehabilitation program adapted to major sensory limitations (Section 2.5, Section 3.1, Section 3.2, Section 3.3 and Section 3.4). The observed changes involved global cognition, executive functioning, verbal learning and memory, functional independence, mobility, longitudinal EEG trajectories, resting-state spectral activity, and sensor-level functional connectivity (Table 2, Table 3, Table 4, Table 5 and Table 6; Figure 2, Figure 3, Figure 4 and Figure 5). These concurrent clinical and neurophysiological changes are described as part of the overall postoperative rehabilitation trajectory and should not be interpreted as evidence of a specific effect of neurofeedback.

Cognitive impairment is common in patients with intracranial meningioma and frequently involves memory, attention, processing speed, and executive functioning [34,35]. Although cognitive performance may improve after surgical resection, postoperative deficits can persist in comparison with healthy individuals and may continue to affect everyday functioning [1,2,3,34,35]. Prospective evidence also indicates that meningioma surgery itself may improve neurocognitive functioning and quality of life, particularly when preoperative dysfunction is related to mass effect or peritumoral edema [36].

In the present case, the MMSE improvement exceeded the published minimal detectable change, the FAB improvement exceeded the threshold for reliable change, and immediate and delayed verbal recall improved reliably across parallel forms (Table 2). Favorable descriptive changes were also observed in working memory, verbal fluency, interference control, processing speed, and several additional cognitive measures (Table 2). These findings are compatible with the postoperative cognitive improvements reported in meningioma cohorts, but the absence of a preoperative neuropsychological assessment prevents separation of surgical recovery from the effects of post-acute rehabilitation [1,2,3,34,36].

Importantly, the patient had already undergone a substantial recovery of consciousness during the acute postoperative phase before admission to intensive neurorehabilitation. However, at rehabilitation admission he continued to show marked neurological, cognitive, behavioral, and functional impairment, including partial disorientation, ideomotor slowing, memory deficits, perseveration, gait ataxia, reduced independence, and a severe dysexecutive profile. The subsequent four-week period therefore represents a subacute phase in which ongoing biological recovery and intensive multimodal rehabilitation occurred concurrently. Although the present uncontrolled design does not allow their respective contributions to be quantitatively disentangled, the observed gains cannot be assumed to reflect spontaneous recovery alone, just as they cannot be attributed specifically to rehabilitation or neurofeedback. Structured early rehabilitation after brain-tumor surgery has previously been associated with meaningful functional and cognitive improvements [4,5,6].

The functional findings are consistent with evidence supporting early rehabilitation after brain-tumor surgery [4]. In a clinical study of postoperative brain-tumor inpatients, a four-week rehabilitation program produced substantial improvements in functional measures, and patients with meningioma showed particularly favorable efficiency in activities of daily living and mobility [4]. Randomized evidence has also shown that structured early cognitive rehabilitation can improve visual attention and verbal memory after primary brain-tumor surgery [6].

Meningioma-specific evidence remains more limited, although a prospective case series using computerized working-memory training reported postoperative neurocognitive improvements that were maintained at three-month follow-up [37]. By contrast, a randomized trial of tablet-based cognitive rehabilitation in patients with glioma or meningioma found high adherence and satisfaction but no significant between-group cognitive or patient-reported benefits, partly in the context of difficult recruitment and limited statistical power [38]. These findings indicate that cognitive rehabilitation is feasible in brain-tumor populations, but its efficacy may depend on timing, delivery format, treatment intensity, and patient selection [6,37,38].

In the present patient, total and cognitive FIM improvements exceeded the published MCID thresholds, whereas the motor FIM improvement did not reach the corresponding threshold and the Modified Barthel Index change remained below the calculated MDC (Table 3). TUG performance improved beyond the published stroke-derived smallest real difference criterion (Table 4). The mixed pattern suggests that the strongest gains involved overall independence, cognition-related autonomy, and mobility efficiency rather than a uniform improvement across all motor and self-care domains (Table 4).

The emotional and behavioral findings were more heterogeneous than the cognitive and functional outcomes (Table 4). Depressive symptoms decreased, and the TAS-20 score shifted from the alexithymic to the non-alexithymic range, whereas state and trait anxiety scores increased slightly (Table 4). Notably, the increase in the total I-DAS score should not be interpreted as evidence of a generalized worsening of apathy, because the domain-specific trajectories were markedly divergent (Table 4; [16]). Executive apathy decreased substantially, whereas emotional apathy and, particularly, initiation apathy increased (Table 4). The improvement in the executive dimension was consistent with the marked gains observed across objective measures of executive control, attention, working memory, verbal fluency, and interference management (Table 2). Conversely, the increase in initiation apathy mainly reflected greater self-reported difficulty in spontaneously generating, planning, and engaging in activities (Table 4; [16]). Given the severe executive dysfunction documented at baseline, reduced awareness of behavioral difficulties during the initial assessment cannot be excluded (Table 2). Accordingly, the apparent increase in initiation apathy may partly reflect improved awareness of previously under-recognized difficulties rather than a true deterioration in everyday behavior; however, this interpretation remains clinical and hypothesis-generating and cannot be demonstrated from the available data (Table 2 and Table 4). These divergent trajectories support the decision to interpret the I-DAS dimensions separately rather than relying exclusively on the total score, because opposing domain-specific changes may be obscured by a global summary score [16]. 

Long-term meningioma cohorts have documented persistent neurocognitive impairment, psychological symptoms, reduced quality of life, and difficulties returning to work even after treatment [39,40]. In one long-term cohort, neurocognitive impairment was particularly evident in visual memory, executive functioning, and attention, while clinically relevant depression and anxiety were also present in substantial patient subgroups [39]. These findings support continued assessment of cognitive, emotional, and functional outcomes rather than relying exclusively on radiological or surgical indicators [39,40].

The longitudinal EEG recordings obtained during the 20 neurofeedback sessions showed a progressive reduction in relative theta activity, increases in SMR and beta activity, an increase in the SMR/theta ratio, and a reduction in the theta/total-beta ratio (Figure 2). These trajectories were obtained using the conservative primary artifact-rejection procedure and were closely reproduced by the less restrictive secondary analysis (Figure 2). The convergence between the two procedures indicates that the principal trajectories were not determined by a small subset of high-amplitude or rapidly varying epochs (Section 3.2).

The increase in the SMR/theta ratio was directionally consistent with the intended training target, which reinforced SMR while inhibiting theta activity (Section 2.5.1 and Section 3.2). However, beta-2 increased proportionally more than SMR, producing a reduction in the SMR/beta-2 ratio and indicating that the spectral evolution was broader than a selective enhancement of SMR alone (Figure 2). The low-gamma increase should remain exploratory because scalp and cranial muscle activity can substantially contaminate high-frequency EEG estimates, particularly above approximately 20–30 Hz [41]. Importantly, the temporal correspondence between these longitudinal EEG trajectories and the neurofeedback sessions should not be interpreted as evidence that neurofeedback specifically caused the observed spectral changes. Because neurofeedback was delivered during a period of active postoperative recovery and concurrent multimodal rehabilitation, the present design cannot disentangle neurofeedback-related learning from other time-varying clinical and rehabilitation-related influences.

The multichannel resting-state recordings provided an independent assessment of PRE–POST neurophysiological change (Table 5; Figure 4). Relative theta power decreased in both eyes-open and eyes-closed conditions, whereas SMR, beta-1, and beta-2 power increased (Table 5). The theta/total-beta ratio decreased markedly, and the SMR/theta ratio increased under both recording conditions (Table 5). The similarity between the longitudinal Cz trajectories and the multichannel resting-state findings indicates descriptive concordance between two independently acquired EEG datasets; however, such concordance does not establish a neurofeedback-specific mechanism or causal treatment effect (Figure 2 and Figure 4).

Resting-state EEG connectivity has previously been associated with cognitive and language functioning in patients with glioma and meningioma [7]. In the present case, theta connectivity decreased in temporal and parietal circuits, posterior alpha connectivity decreased particularly during eyes-closed recording, and SMR connectivity increased across several fronto-central and parieto-occipital connections (Table 6; Figure 5). These findings indicate frequency-specific PRE–POST differences in sensor-level connectivity rather than a uniform increase or decrease in global synchronization (Table 6; Figure 5).

The wPLI reduces sensitivity to instantaneous zero-lag coupling and is therefore less affected than conventional coherence by common sources and volume conduction [31]. However, scalp-level wPLI does not completely eliminate volume-conduction effects and cannot identify the cortical generators underlying the observed regional changes [31]. Accordingly, the connectivity findings should be interpreted as descriptive sensor-level changes rather than direct evidence of restored network efficiency or normalization [31].

The present neurophysiological findings are broadly compatible with previous preliminary reports of neurofeedback in patients with frontal brain lesions and postoperative brain-tumor populations [8,9,42]. A randomized double-blind study of infra-low-frequency neurofeedback in patients with acquired frontal brain lesions did not demonstrate a clear overall cognitive advantage over sham treatment, although exploratory subgroup analyses suggested possible benefits in patients with predominantly frontal lesions [8].

A previous case report involving a patient who underwent resection of a tuberculum sellae meningioma described improvements in attention, executive functioning, processing speed, and frontal qEEG measures after an intensive neurofeedback program [42]. A subsequent prospective case series of seven postoperative brain-tumor inpatients reported that 15 neurofeedback sessions delivered alongside motor rehabilitation were feasible and well tolerated, with cognitive improvements in all participants and heterogeneous changes in qEEG, mood, motor performance, and activities of daily living [9].

The wider literature remains mixed and does not establish neurofeedback-specific efficacy in neuro-oncology [43]. A systematic review of cognitive rehabilitation in brain-tumor survivors identified promising results for several structured cognitive interventions but highlighted heterogeneity in populations, protocols, outcome measures, and study quality [43]. An exploratory randomized trial in patients with non-central nervous system cancers found improvements in affective symptoms after neurofeedback but no clear benefit for perceived cognitive impairment [44]. These findings support the characterization of neurofeedback as a feasible and potentially useful adjunct rather than an established standalone treatment for postoperative cognitive impairment [8,9,42,43,44].

The auditory adaptation of neurofeedback was clinically relevant because the patient’s severe visual impairment limited the usefulness of conventional visually mediated feedback (Section 2.5.1). Cognitive exercises were similarly adapted through verbal presentation, auditory material, high-contrast stimuli, and progressive adjustment of task difficulty (Section 2.5.2). The patient completed all 20 sessions and tolerated increasing cognitive demands while maintaining high accuracy on several trained tasks (Section 3.3; Figure 3). Previous case-level and case-series evidence also indicates that intensive neurofeedback can be implemented feasibly during inpatient neurorehabilitation after brain-tumor surgery [9,42].

Beyond meningioma, this sensory-adapted approach may also be applicable to selected patients undergoing rehabilitation after resection of other intracranial tumors, including gliomas and brain metastases, particularly when auditory pathways and auditory comprehension are sufficiently preserved to support reliable engagement with auditory feedback. This may be especially relevant in patients considered suitable for intraoperative functional monitoring or awake craniotomy, in whom preserved communication and active task participation are central to functional assessment during surgery. Awake craniotomy is an established approach for tumors involving or adjacent to eloquent cortical regions, and both asleep–awake–asleep and monitored anesthesia care techniques have been systematically evaluated in this setting [45]. Accordingly, auditory EEG neurofeedback may represent a flexible postoperative rehabilitation adjunct in carefully selected neuro-oncological patients when visual feedback is limited or impractical; however, this broader applicability remains hypothetical and requires prospective investigation.

Several limitations must be acknowledged. First, the single-case design and absence of a control condition prevent causal attribution of the observed changes to the rehabilitation program or to neurofeedback specifically. Second, the intervention combined neurofeedback, cognitive training, physiotherapy, routine clinical care, and postoperative recovery, making it impossible to isolate the contribution of each component. Third, the short interval from surgery to rehabilitation means that spontaneous recovery, reduction in edema, and relief of mass effect were likely contributors to the observed cognitive and functional changes [1,34,36].

Fourth, several RCI, MCID, and MDC thresholds were derived from populations other than patients with brain tumors and were therefore used only as indirect supportive criteria (Section 2.7.1). Fifth, some visually mediated neuropsychological measures may have remained sensitive to the patient’s severe visual impairment despite clinical adaptation. Sixth, single-channel neurofeedback-session EEG did not permit ICA, spatial source separation, or multichannel artifact correction, and the beta-2 and low-gamma results remain potentially sensitive to residual myogenic activity [41].

Seventh, the resting-state analyses were conducted at sensor level, and structural distortion associated with the tumor, surgery, edema, and postoperative changes limits anatomical interpretation of the scalp topographies and connectivity maps. Eighth, only a single pre-intervention resting-state EEG assessment was available; therefore, baseline stability and normal intra-individual variability in spectral and connectivity measures could not be estimated. Ninth, the study did not include delayed neuropsychological, functional, or EEG follow-up, preventing assessment of the durability of the observed changes. This limitation is clinically important because long-term cognitive, psychological, and occupational difficulties can persist in meningioma survivors [39,40].

Despite these limitations, the successful completion of all 20 neurofeedback sessions despite severe visual impairment, together with the acquisition of analyzable longitudinal and resting-state EEG data, supports the practical feasibility of integrating sensory-adapted EEG neurofeedback within a broader postoperative rehabilitation program (Table 2, Table 3, Table 4, Table 5 and Table 6; Figure 2, Figure 3, Figure 4 and Figure 5). The findings should be interpreted as hypothesis-generating rather than as evidence of treatment-specific efficacy [8,9,42,43,44]. Controlled studies with larger samples, rehabilitation-only comparison groups, repeated follow-up assessments, standardized neuro-oncological outcomes, and source-level electrophysiological analyses are required to determine whether neurofeedback provides benefits beyond those attributable to surgery, spontaneous recovery, and conventional rehabilitation [8,34,35,43]. More specifically, future studies could incorporate repeated pre-intervention assessments, where clinically feasible, to better characterize postoperative recovery trajectories and intra-individual EEG variability. A subsequent step should compare standard multimodal rehabilitation with the same rehabilitation program supplemented by auditory EEG neurofeedback, ideally within randomized controlled designs, while also incorporating session-by-session neurofeedback learning indices and delayed clinical, neuropsychological, and electrophysiological follow-up [8,9,43].

## 5. Conclusions

This case report describes the feasibility of an intensive, sensory-adapted multimodal neurorehabilitation program following emergency resection of a giant olfactory groove meningioma. The patient completed 20 sessions combining auditory EEG neurofeedback, individualized cognitive training, and physiotherapy despite severe bilateral visual impairment and marked baseline executive dysfunction.

The rehabilitation period was accompanied by improvements in global cognition, executive functioning, verbal learning and memory, functional independence, and mobility, with several changes exceeding the predefined reliable-change or published measurement-based thresholds. Longitudinal EEG recordings obtained during neurofeedback showed reduced theta activity, increased SMR and beta activity, increased SMR/theta, and reduced theta/total-beta. Multichannel resting-state EEG showed lower theta activity and higher SMR and beta activity under both recording conditions, together with frequency-specific PRE–POST differences in sensor-level functional connectivity.

The observed clinical and neurophysiological changes cannot be attributed specifically to neurofeedback because the intervention was multimodal and was delivered during a period of active postoperative recovery. Surgery, reduction in mass effect and edema, cognitive training, physiotherapy, medication, sleep and arousal changes, and spontaneous recovery may all have contributed to the findings. The absence of a control condition and delayed follow-up further limits causal and long-term interpretation.

Nevertheless, this case supports the practical integration of auditory EEG neurofeedback into individualized inpatient rehabilitation when visual feedback is not feasible and suggests its potential applicability to selected patients undergoing rehabilitation after surgery for other brain tumors, including gliomas and brain metastases, when auditory pathways and comprehension are sufficiently preserved. Prospective controlled studies with larger samples, rehabilitation-only comparison groups, standardized neuro-oncological outcomes, repeated follow-up assessments, and source-level EEG analyses are required to determine whether neurofeedback provides additional benefits beyond conventional rehabilitation and natural postoperative recovery.

## Figures and Tables

**Figure 1:**
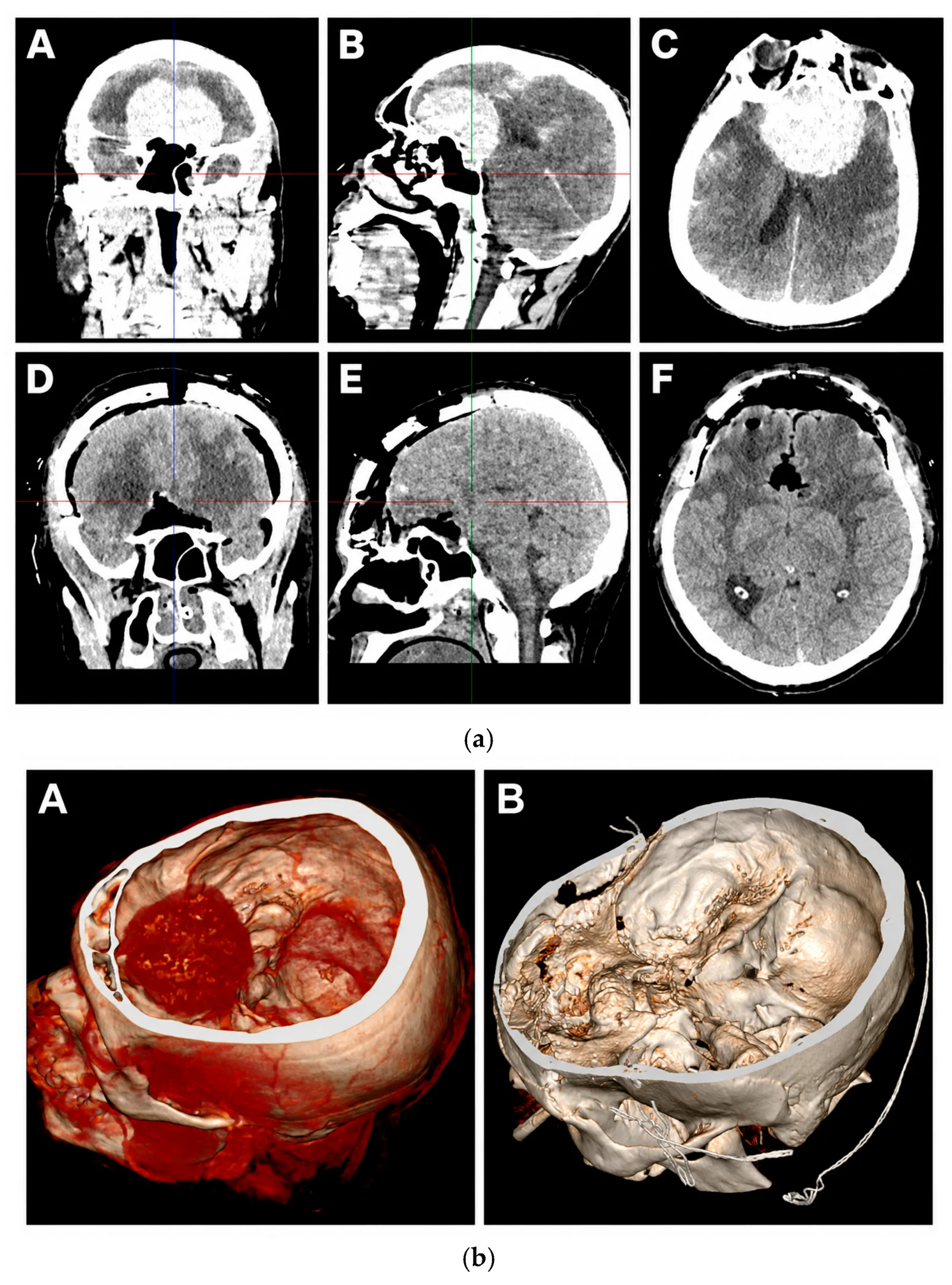
(**a**). Pre- and postoperative computed tomography (CT) imaging. (**A**–**C**) Preoperative contrast-enhanced CT images in the coronal, sagittal, and axial planes showing a giant calcified extra-axial olfactory groove meningioma extending toward the planum sphenoidale and chiasmatic region, with marked bilateral frontal-lobe compression, extensive peritumoral edema, and substantial mass effect. (**D**–**F**) Early postoperative CT images in approximately corresponding coronal, sagittal, and axial planes obtained after surgical resection, showing the resection cavity and expected postoperative changes, including intracranial air, with marked reduction in the preoperative mass effect. (**b**). Three-dimensional computed tomography (CT) reconstructions. (**A**) Preoperative 3D CT reconstruction showing the giant extra-axial olfactory groove meningioma occupying the anterior cranial fossa, with extensive involvement of the frontal base and marked mass effect on the adjacent frontal structures. (**B**) Postoperative 3D CT reconstruction after surgical resection, showing removal of the tumor mass with decompression of the anterior cranial fossa and expected postoperative changes.

**Figure 2:**
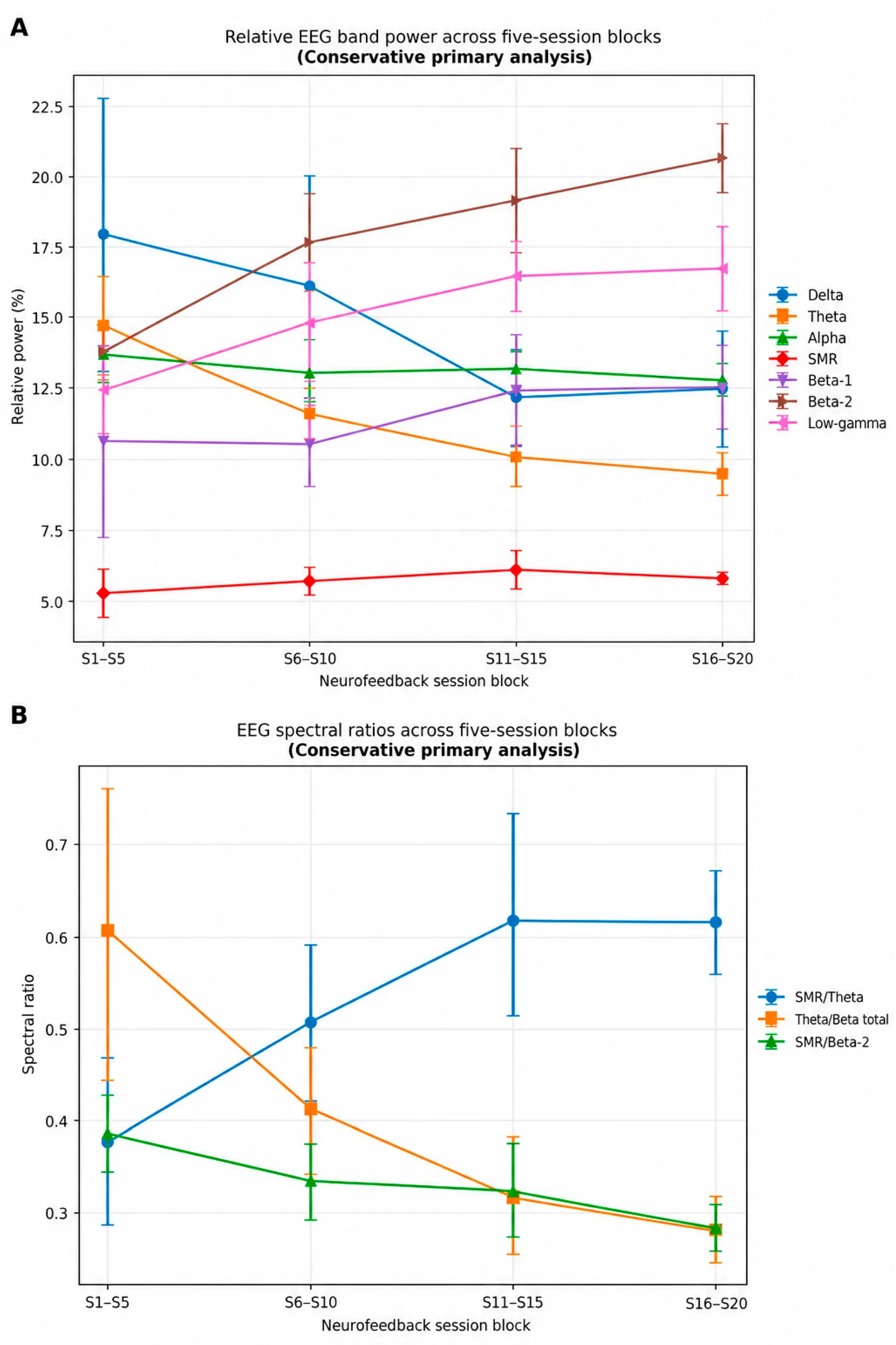
Longitudinal EEG dynamics across the 20 neurofeedback sessions based on the conservative primary analysis. Sessions were grouped into four consecutive five-session blocks: S1–S5, S6–S10, S11–S15, and S16–S20. (**A**) Relative power in the delta, theta, alpha, sensorimotor rhythm (SMR), beta-1, beta-2, and low-gamma frequency bands. (**B**) SMR/theta, theta/total-beta, and SMR/beta-2 spectral ratios. Values represent the mean ± standard deviation of the five session-level median values included in each block after application of the conservative primary epoch-level artifact-rejection criteria.

**Figure 3:**
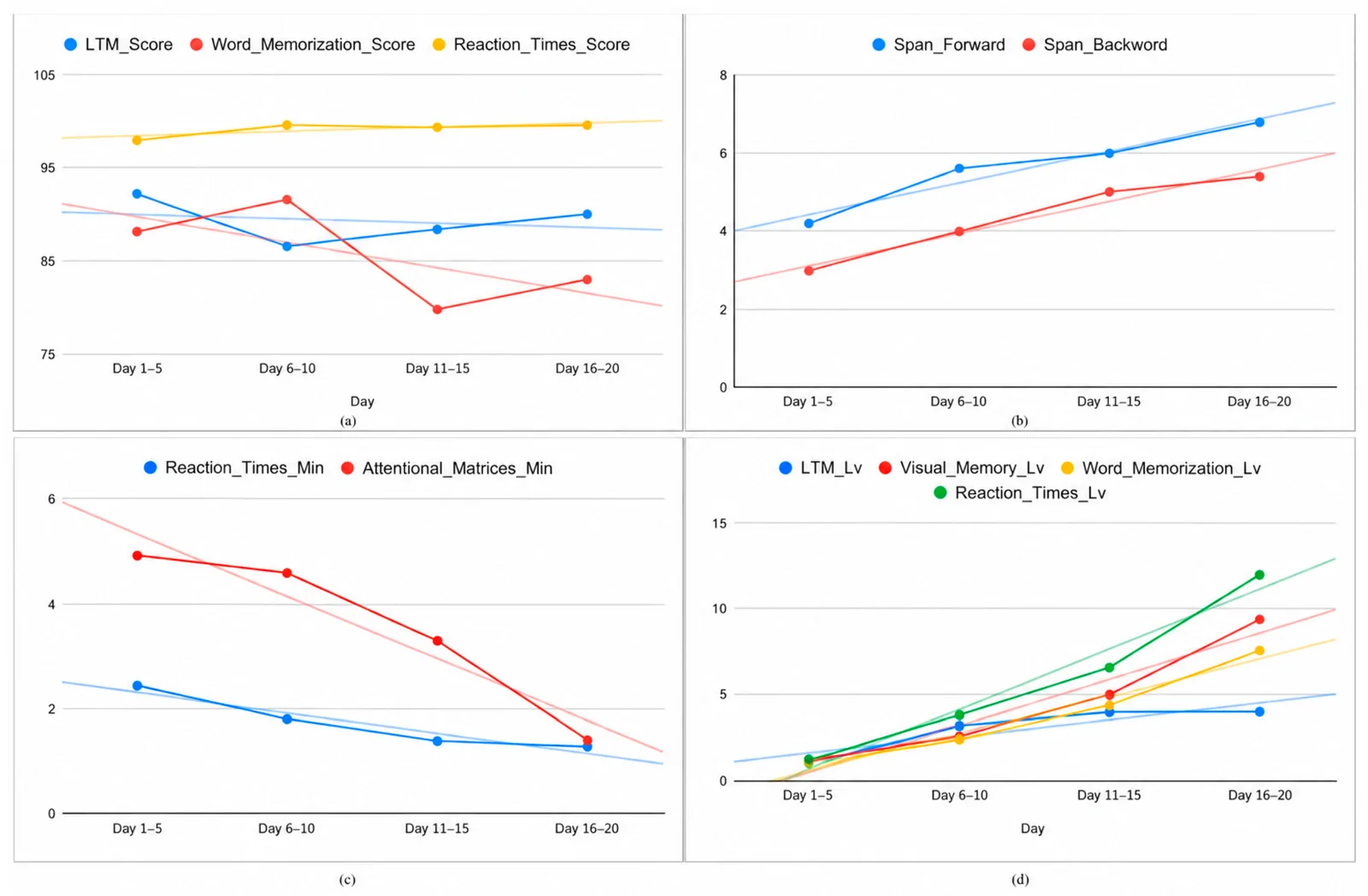
Cognitive training outcomes across the 20 rehabilitation sessions, grouped into 5-day blocks. (**a**) Accuracy scores for Long-Term Memory (MLT), Word Memorization, and Reaction Time tasks. (**b**) Progressive expansion of verbal working memory capacity (Forward and Backward Span). (**c**) Reduction in execution times (in minutes) for Reaction Time and Attentional Matrices tasks. (**d**) Gradual escalation of task difficulty levels (Lv) accommodated by the patient across different training modules. Lighter lines represent linear trend lines fitted across the four consecutive session blocks.

**Figure 4:**
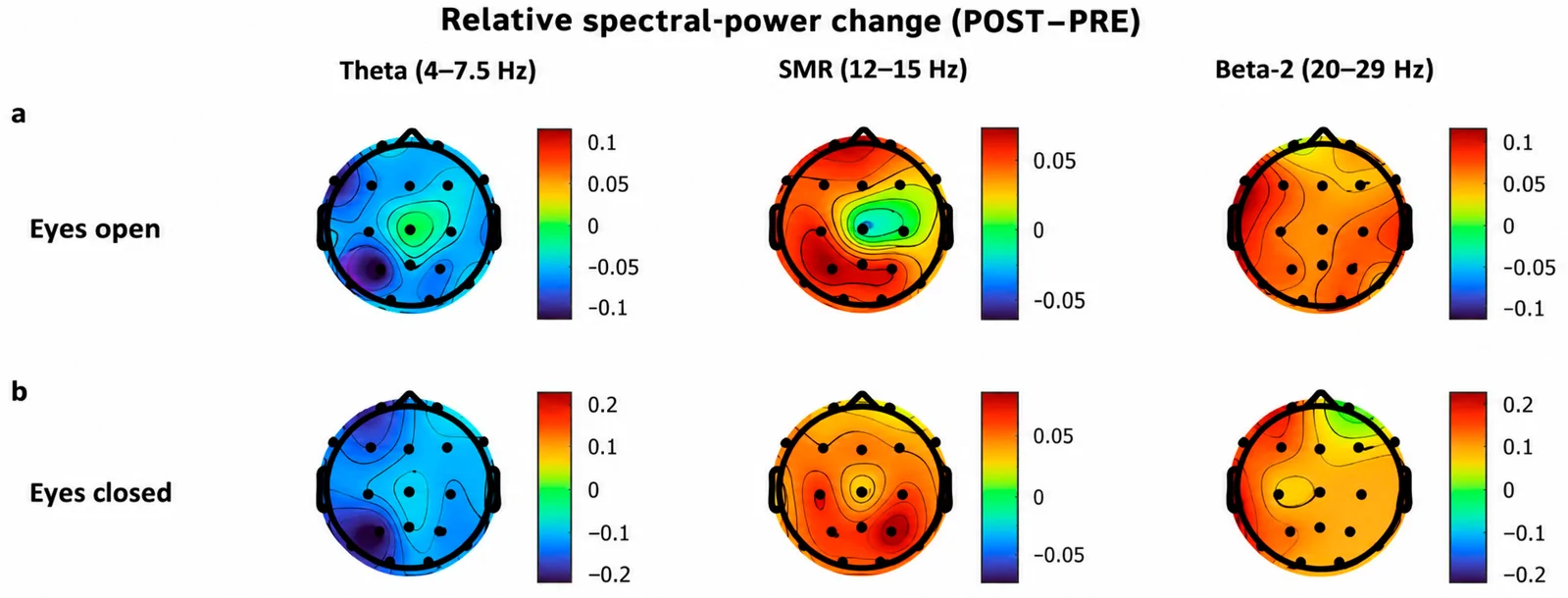
Selected topographic changes in resting-state relative spectral power after the rehabilitation program. Topographic maps show the POST–PRE change in relative spectral power for the three frequency bands most directly related to the main neurophysiological findings: theta (4–7.5 Hz), sensorimotor rhythm (SMR, 12–15 Hz), and beta-2 (20–29 Hz). Changes are displayed separately for the eyes-open (**a**) and eyes-closed (**b**) conditions. Negative values indicate a post-treatment reduction in relative power, whereas positive values indicate an increase. All 19 electrodes were retained for scalp visualization after spherical interpolation of previously excluded noisy channels; however, quantitative PRE–POST spectral comparisons were calculated using matched non-interpolated channels only.

**Figure 5:**
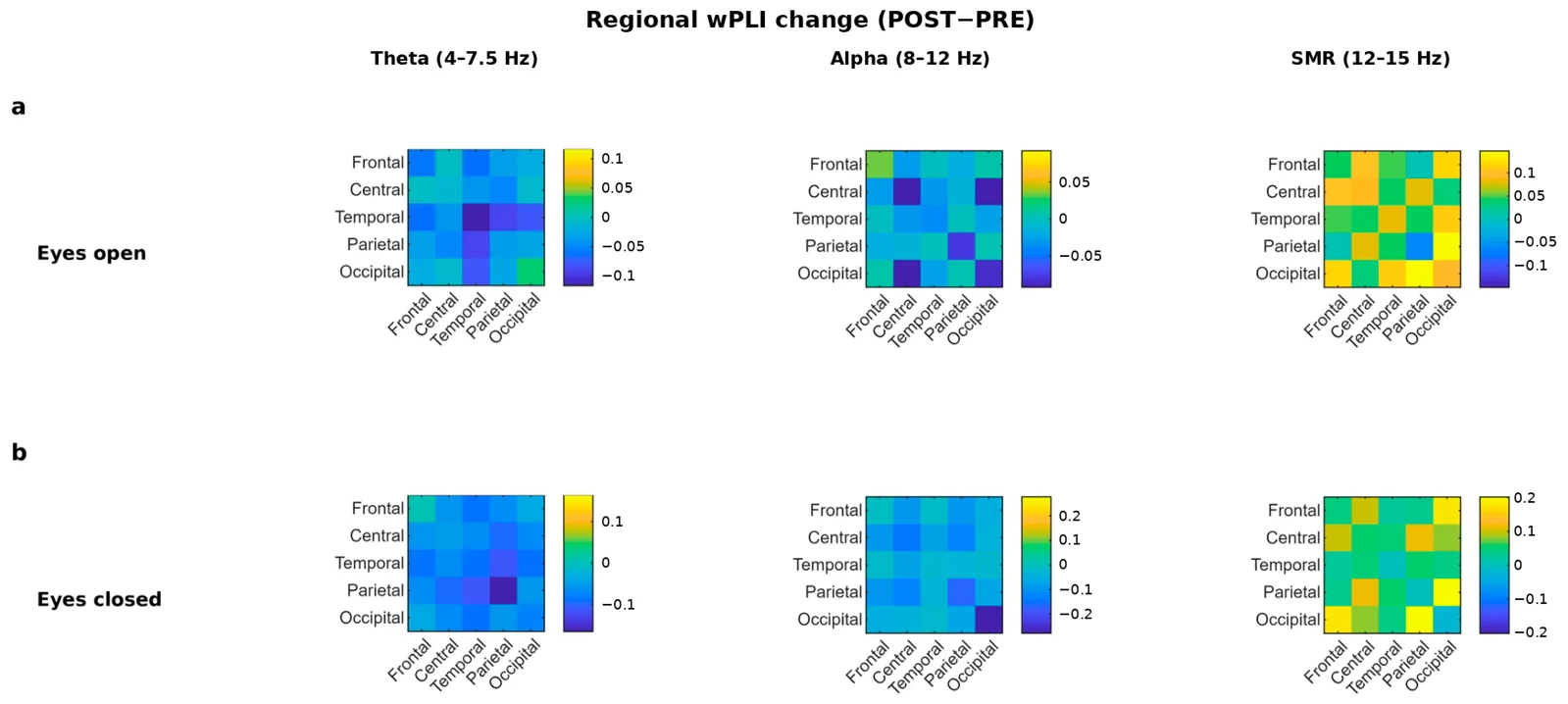
Selected regional changes in resting-state functional connectivity after the rehabilitation program. Regional heatmaps show the POST–PRE change in weighted phase-lag index (wPLI) for theta (4–7.5 Hz), alpha (8–12 Hz), and sensorimotor rhythm (SMR, 12–15 Hz), separately for the eyes-open (**a**) and eyes-closed (**b**) conditions. Rows and columns represent the frontal, central, temporal, parietal, and occipital regions. Positive values indicate increased post-treatment phase-based connectivity, whereas negative values indicate decreased connectivity. Connectivity estimates were calculated only from electrode pairs that did not include interpolated channels.

**Table 1:** Overview of the clinical features.

Clinical Case Features Overview	
Age	53 years old
Gender	Male
Medical History and Physical Examination	Keratoconus, untreated depressive state (following bereavement)
Neurological Assessment	Generalized four-limb weakness (MRC 4/5), gait ataxia, severe visual impairment, bilateral quadriceps hypotrophy
Cognitive Assessment	Mild-to-moderate global cognitive impairment (MMSE 23), profound dysexecutive syndrome, severe inhibitory and processing speed deficits, impaired verbal and working memory
Motor Assessment	Reduced postural control, limited autonomy in transfers, impaired trunk stability (TCT = 45), decreased functional independence (Modified Barthel Index = 66), elevated care complexity (RCS-E = 8)
Pharmacological Treatment	Levetiracetam (Keppra) 500 mg three times daily; Enoxaparin (Clexane) 4000 IU once daily; Pantoprazole 40 mg once daily; Dexamethasone (Soldesam oral drops, 2 mg/mL [0.2%]) 64 drops once daily

**Table 2:** Pre–post neuropsychological outcomes and individual-level change interpretation.

Measure	Pre	Post	Raw Change	Change Interpretation
MMSE	23	29	+6	Exceeded MDC95 (2.84-point threshold)
FAB	5	17	+12	Reliable improvement (RCI = 12.17; |RCI| ≥ 1.96)
Clock Drawing Test	2	9	+7	Descriptive improvement; formal index not calculated
Prose recall, immediate	3.6	4.6	+1.0	Descriptive improvement; formal index not calculated
Prose recall, delayed	0	5.9	+5.9	Descriptive improvement; formal index not calculated
RAVLT/RWL, immediate	26	41	+15	Reliable improvement (ZD = 3.27; |ZD| ≥ 1.65)
RAVLT/RWL, delayed	4	8	+4	Reliable improvement (ZD = 2.98; |ZD| ≥ 1.65)
Digit Span Forward	3	6	+3	Descriptive improvement; formal index not calculated
Digit Span Backward	2	6	+4	Descriptive improvement; formal index not calculated
Weigl’s Sorting Test	0	7	+7	Descriptive improvement; formal index not calculated
Phonemic fluency	4	26	+22	Descriptive improvement; formal index not calculated
Semantic fluency	21	42	+21	Descriptive improvement; formal index not calculated
Spatial Stroop, time	261	172	−89	Descriptive reduction in execution time
Spatial Stroop, errors	13	1	−12	Descriptive reduction in errors
Hayling Section A, time (s)	106.53	40.35	−66.18	Descriptive improvement; from severely slowed to within normative range
Hayling Section B, time (s)	214.35	134.23	−80.12	Descriptive improvement; from severely slowed to within normative range
Hayling Section B, total errors	10/15	2/15	−8	Descriptive reduction in inhibition errors
Hayling Section B, correct responses	5/15	13/15	+8	Descriptive improvement from deficient to normative performance

Notes: Positive raw change denotes improvement except for completion-time and error measures, for which lower values are better. The FAB RCI was calculated using the Italian normative standard deviation and test–retest reliability reported by Appollonio et al. [11]. RAVLT ZD values were calculated for parallel forms using Carlesimo et al. [14]; sensitivity analyses across all possible ordered form pairs yielded the same classification. The MMSE was compared with the published MDC95 [33]. No formal change index was calculated when the exact version, scoring system, or directly applicable test–retest parameters could not be verified. Hayling Sentence Completion Test outcomes were interpreted descriptively according to the normative framework of the Italian version [12]; no formal reliable-change index was calculated.

**Table 3:** Pre–post functional and mobility outcomes.

Measure	Pre	Post	Raw Change	Measurement-Based Interpretation
FIM total	80	110	+30	MCID reached (22-point threshold)
FIM motor	65	80	+15	MCID not reached (17-point threshold)
FIM cognitive	15	30	+15	MCID reached (3-point threshold)
Modified Barthel Index	66	72	+6	MDC% not reached (individual threshold = 13.11 points)
Timed Up and Go, s	18.35	9.11	−9.24	Reliable improvement (95% SRD improvement limit: Δ < −3.75 s)

Notes: For the TUG, a negative raw change indicates improvement because shorter completion time is better. FIM thresholds are anchor-based MCIDs reported in post-stroke rehabilitation [20]. For the Modified Barthel Index, the 19% MDC was applied to the participant-specific mean of the two observations because the source identified heteroscedastic measurement error [21]. For the TUG, the original 95% smallest real difference limits were used [22]. These stroke-derived benchmarks were treated as indirect supportive criteria rather than neuro-oncology-specific thresholds.

**Table 4:** Pre–post emotional and behavioral outcomes.

Measure	Pre	Post	Raw Change	Descriptive Interpretation
BDI	9	0	−9	Reduction in depressive symptom score
STAI-Y1	36	40	+4	Slight increase in state anxiety score
STAI-Y2	38	40	+2	Slight increase in trait anxiety score
TAS-20	66	39	−27	Shift from alexithymic to non-alexithymic range
I-DAS total	26	32	+6	Net increase in total score despite divergent subscale changes
I-DAS executive	14	3	−11	Marked reduction in executive-apathy score
I-DAS emotional	9	13	+4	Increase in emotional-apathy score
I-DAS initiation	3	16	+13	Marked increase in self-reported initiation-apathy score

Notes: Higher BDI, STAI, TAS-20, and I-DAS scores indicate greater symptom burden. Emotional and behavioral outcomes were interpreted descriptively because no directly applicable RCI, MCID, or MDC was verified for the specific versions used. TAS-20 cut-offs are categorical and do not represent a reliable-change or clinically important difference threshold [15]. I-DAS dimensions were interpreted separately because the subscales showed divergent trajectories [16].

**Table 5:** Whole-scalp resting-state relative spectral power and spectral ratios calculated from matched non-interpolated channels.

Measure	OA PRE	OA POST	OA Change, %	OC PRE	OC POST	OC Change, %
Delta relative power, %	23.90	19.52	−18.3	23.15	16.93	−26.9
Theta relative power, %	19.79	12.85	−35.1	22.85	9.32	−59.2
Alpha relative power, %	21.89	17.36	−20.7	23.83	19.21	−19.4
SMR relative power, %	6.26	9.46	+51.0	5.00	9.78	+95.5
Beta-1 relative power, %	8.79	13.93	+58.5	6.76	13.93	+106.3
Beta-2 relative power, %	5.64	11.80	+109.1	4.56	13.26	+190.9
Low-gamma relative power, %	4.09	8.72	+113.1	3.52	11.52	+227.1
Theta/total-beta ratio	1.715	0.536	−68.7	2.507	0.380	−84.9
SMR/theta ratio	0.333	0.799	+140.2	0.225	1.144	+407.5

**Note:** OA, eyes open; OC, eyes closed; SMR, sensorimotor rhythm. Relative power represents the percentage of total 1–45 Hz power. Change percentages represent proportional change relative to the PRE value rather than percentage-point differences. OA estimates were calculated from 17 matched non-interpolated channels; OC estimates were calculated from 18 matched non-interpolated channels. Low-gamma outcomes should be interpreted cautiously because of their potential residual sensitivity to myogenic activity.

**Table 6:** Selected regional changes in resting-state weighted phase-lag index.

Condition	Band	Regional Connection	Pair Type	Pairs, *n*	PRE wPLI	POST wPLI	ΔwPLI
OA	Theta	Temporal–Temporal	Within	3	0.267	0.063	−0.204
OA	Theta	Temporal–Parietal	Between	9	0.171	0.048	−0.123
OA	SMR	Parietal–Occipital	Between	6	0.068	0.216	+0.147
OA	SMR	Frontal–Occipital	Between	12	0.050	0.158	+0.108
OA	Low-gamma	Temporal–Temporal	Within	3	0.035	0.189	+0.154
OA	Low-gamma	Central–Central	Within	3	0.074	0.203	+0.129
OA	Low-gamma	Central–Temporal	Between	9	0.039	0.148	+0.109
OC	Alpha	Occipital–Occipital	Within	1	0.283	0.005	−0.278
OC	Alpha	Parietal–Parietal	Within	3	0.240	0.074	−0.166
OC	Alpha	Central–Central	Within	3	0.220	0.075	−0.144
OC	Theta	Parietal–Parietal	Within	3	0.208	0.043	−0.164
OC	Theta	Temporal–Parietal	Between	9	0.195	0.049	−0.146
OC	Theta	Temporal–Temporal	Within	3	0.214	0.089	−0.126
OC	SMR	Parietal–Occipital	Between	6	0.079	0.281	+0.202
OC	SMR	Frontal–Occipital	Between	14	0.043	0.220	+0.177
OC	SMR	Central–Parietal	Between	9	0.102	0.215	+0.114
OC	SMR	Frontal–Central	Between	21	0.067	0.165	+0.098

**Note:** OA, eyes open; OC, eyes closed; SMR, sensorimotor rhythm; wPLI, weighted phase-lag index. Values represent the mean across the available non-interpolated electrode pairs assigned to each regional connection. ΔwPLI was calculated as POST minus PRE. The occipital–occipital estimate corresponds to the single O1–O2 electrode pair. Low-gamma connectivity findings were considered exploratory because residual myogenic contributions cannot be completely excluded.

## Data Availability

The data presented in this study are available on request from the corresponding author. The data are not publicly available due to privacy and ethical restrictions.
